# Supplementary material for: Context-specific regulatory genetic variation in MTOR dampens neutrophil-T cell crosstalk in pneumonia-associated sepsis
Source: Nat Commun. 2026 Feb 25;17:3201. doi: 10.1038/s41467-026-69919-7 (PMC13057173; doi:10.1038/s41467-026-69919-7)
Supplement: Supplementary file 1 — Supplementary Information [file 41467_2026_69919_MOESM1_ESM.pdf]

Supplementary Information

Context-specific regulatory genetic variation in *MTOR* dampens neutrophil-T cell crosstalk in pneumonia-associated sepsis

Ping Zhang[1,2*], Patrick MacLean[2], Alicia Jia[2], Callum R. O'Neill[2], Alice Allcock[2], Ethan Prince[2], Bora Ozcan[3], Roman M. Doll[3], Imogen Dyne[2], Kiki Cano-Gamez[2,4], Hanyu Qin[1,2], Chloe Wainwright[2], Giuseppe Scozzafava[2], Andrew C. Brown[2], James O. J. Davies[3,5], Amanda Y. Chong[2], Alexander J. Mentzer[1,2], Katie L. Burnham[6], Emma E. Davenport[6], and Julian C. Knight[1,2,5*]

[1]Chinese Academy of Medical Sciences Oxford Institute, Nuffield Department of Medicine, University of Oxford, Oxford, UK.
[2]Centre for Human Genetics, Nuffield Department of Medicine, University of Oxford, Oxford, UK.
[3]MRC Molecular Haematology Unit, MRC Weatherall Institute of Molecular Medicine, Radcliffe Department of Medicine, University of Oxford, Oxford, UK
[4]Department of Clinical and Biomedical Sciences, University of Exeter, Exeter, UK
[5]Oxford National Institute of Health Research Biomedical Research Centre, University of Oxford, Oxford, UK
[6]Wellcome Sanger Institute, Wellcome Genome Campus, Hinxton, Cambridge, UK

*to whom correspondence should be addressed (julian.knight@well.ox.ac.uk or ping.zhang@well.ox.ac.uk)

Supplementary Figures 1-10

Supplementary Fig. 1. Linkage disequilibrium structure and eQTL associations at the *MTOR* locus, with interaction involving NLR, MLR and sepsis response signatures.

Supplementary Fig. 2. Genetic and clinical associations of 28-day mortality with clinical phenotypes including MLR, FiO_2 levels and the *MTOR* eQTL rs4845987 with or without stratification by cancer status, T2D or HbA1c levels.

Supplementary Fig. 3. Population genetics and cross-trait colocalisation of *MTOR* eQTL with T2D GWAS loci, showing ancestry-specific allele frequencies and Fst-based population differentiation across global cohorts.

Supplementary Fig. 4. Flow cytometry analysis of neutrophil-T cell co-culture experiments showing activation-induced expression of CD64, CD123, and PD-L1 on sepsis neutrophils and corresponding transcriptional changes based on scRNA-seq analysis.

Supplementary Fig. 5. Functional readout of neutrophil activation and NETosis upon T cell interaction, and rapamycin-dependent modulation of activation marker expression on sepsis neutrophils.

Supplementary Fig. 6. *Ex vivo* sepsis whole-blood co-culture experiments showing neutrophil and T cell activation in response to T cell conditioned media or anti-CD3/CD28 stimulation, quantified by flow cytometry.

Supplementary Fig. 7. Epigenomic profiling of the *MTOR* locus highlighting regulatory variants within T cell ATAC-seq peaks, and eQTL activity across $CD4^+$ T cell subsets under different activation conditions.

Supplementary Fig. 8. Epigenomic profiling of the *MTOR* locus showing histone marks, chromatin looping, and DNA hydroxymethylation changes upon T cell activation.

Supplementary Fig. 9. Functional validation of *MTOR* siRNA and base editing experiments confirming transcript silencing and precise genomic targeting.

Supplementary Fig. 10. Cell type deconvolution analysis via CIBERSORTx showing differences in cell absolute scores between SRS1 and non-SRS1 sepsis groups.

Supplementary Figure 1

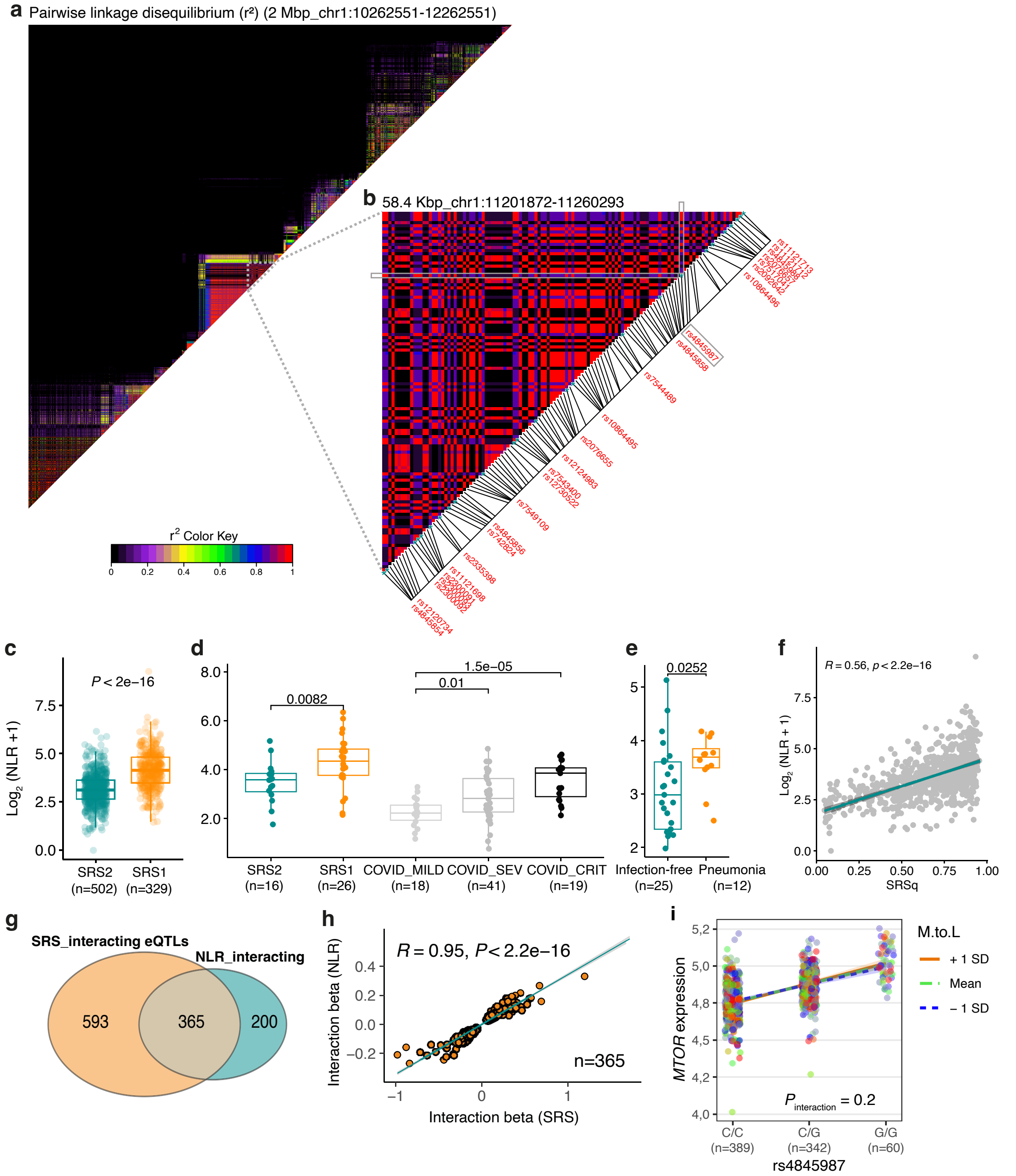

Supplementary Fig. 1. (a) Pairwise linkage disequilibrium (r^2) for SNPs tested for eQTL association at the *MTOR* gene locus (±1 Mbp from the *MTOR* transcription start site). r^2 was calculated using PLINK with GAinS genotype data (n=1,168) and visualised with LDheatmap. **(b)** A zoomed-in view of the locus containing the 25 *MTOR* lead eQTL variants (red text). rs4845987 is highlighted within the grey square. (**c-e**) Box plots showing the heightened N-to-T ratios are associated with severe disease. Cell counts from sepsis and COVID-19 patients were retrieved from GAinS cohort (c) and *COMBAT Consortium*[1] (d). 48h post-operative pneumonia data was obtained from *Torrance & Zhang et al*[2] (e). (**f**) Correlation of log2 NLR and SRSq in samples derived from sepsis patients of the UK GAinS cohort (n=831). Pearson's r and *p* values are shown. (**g**) Venn diagram showing the overlap of SRS-interacting and NLR-interacting eQTLs identified in sepsis patients (n=638). (**h**) Correlation of effect sizes of the shared SRS- and NLR-interacting eQTL associations. (**i**) Interaction of monocyte-to-lymphocyte ratio (MLR) with the MTOR eQTL association in whole blood. P value was obtained from a linear mixed model with log2-transformed MLR, using the default Satterthwaite's approximation for degrees of freedom as implemented in the R package lmerTest.

Supplementary Figure 2

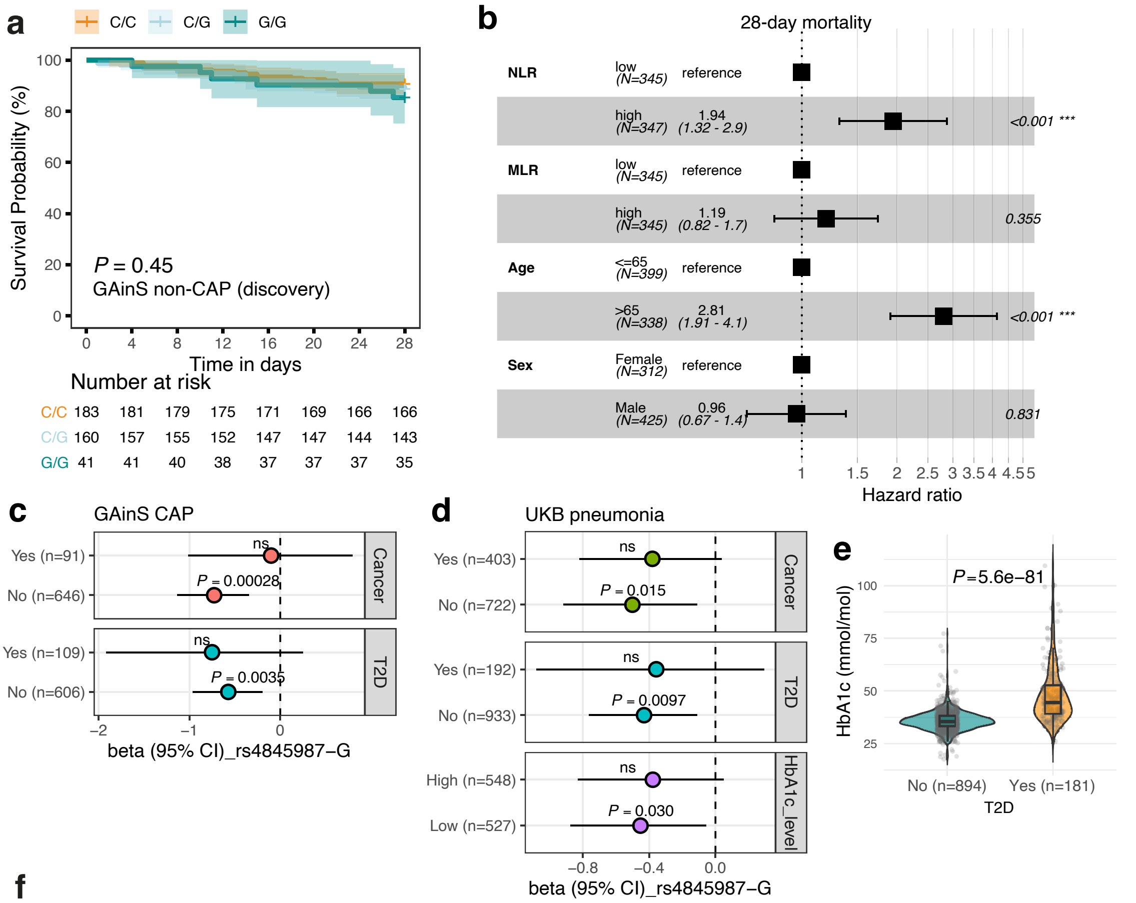

f

	GAinS				GenOSept		
**Univariable Cox model analysis**	n	**HR (95% CI)**	**p value**		n	**HR (95% CI)**	**p value**
severe (FiO2 ≥0.4) vs moderate/mild	372 vs 351	2.649 (1.811-3.876)	5.23E-07		360 vs 277	2.625 (1.659-4.154)	3.77E-05
severe (0< PaO2/FiO2 ≤100) vs moderate/mild	88 vs 493	3.809 (2.593-5.596)	9.38E-12		256 vs 330	1.441 (0.9673-2.145)	0.0724
Age: >65 years *vs* ≤65 years	338 vs 399	2.705 (1.882-3.888)	7.61E-08		313 vs 356	2.684 (1.779-4.05)	2.53E-06
Sex: male *vs* female	425 vs 312	1.123 (0.7942-1.587)	0.512		440 vs 229	0.8481 (0.5726-1.256)	0.411
**Multivariable Cox model analysis**							
severe (FiO2 ≥0.4) vs moderate/mild	372 vs 351	1.9321 (1.2066-3.094)	6.11E-03		360 vs 277	2.7128 (1.6821-4.375)	4.27E-05
severe (0< PaO2/FiO2 ≤100) vs moderate/mild	88 vs 493	2.9327 (1.9295-4.458)	4.73E-07		256 vs 330	1.37 (0.9174-2.046)	0.124
Age: >65 years *vs* ≤65 years	338 vs 399	2.3302 (1.5935-3.407)	1.28E-05		313 vs 356	2.6019 (1.7015-3.979)	1.02E-05
Sex: male *vs* female	425 vs 312	0.9815 (0.6751-1.427)	9.22E-01		440 vs 229	0.7568 (0.5026-1.140)	0.182

Supplementary Fig. 2. (**a**) Kaplan–Meier curves for 28-day mortality in sepsis non-CAP patients of GAinS (n= 384) cohort with different alleles of the *MTOR* eQTL rs4845987. *P* value was calculated using cox regression adjusted for age and gender. (**b**) Multivariable Cox regression analysis of NLR and MLR in relation to 28-day sepsis mortality in the GAinS CAP cohort. (**c-d**) Forest plots showing 28-day mortality for GAinS-CAP (c) or UKB pneumonia (d) patients carrying the G alleles, stratified according to all-cause cancer status, T2D status or HbA1c levels (see **Methods**). Beta coefficient and P value was calculated using additive logistic regression, adjusting for age, gender and the first seven genotype principal components from Europeans. (**e**) Violin plot of HbA1c levels in UKB pneumonia patients without T2D (cyan) or with T2D (Orange). P value was calculated using linear regression adjusting for age and gender. Data missing for 10 patients in the UKB without HbA1c measurements. (**f**) Cox regression analysis for hypoxia-related prognostic factors associated with sepsis 28-day mortality in GAinS (left) and GenOSept (right) sepsis CAP cohorts.

Supplementary Figure 3

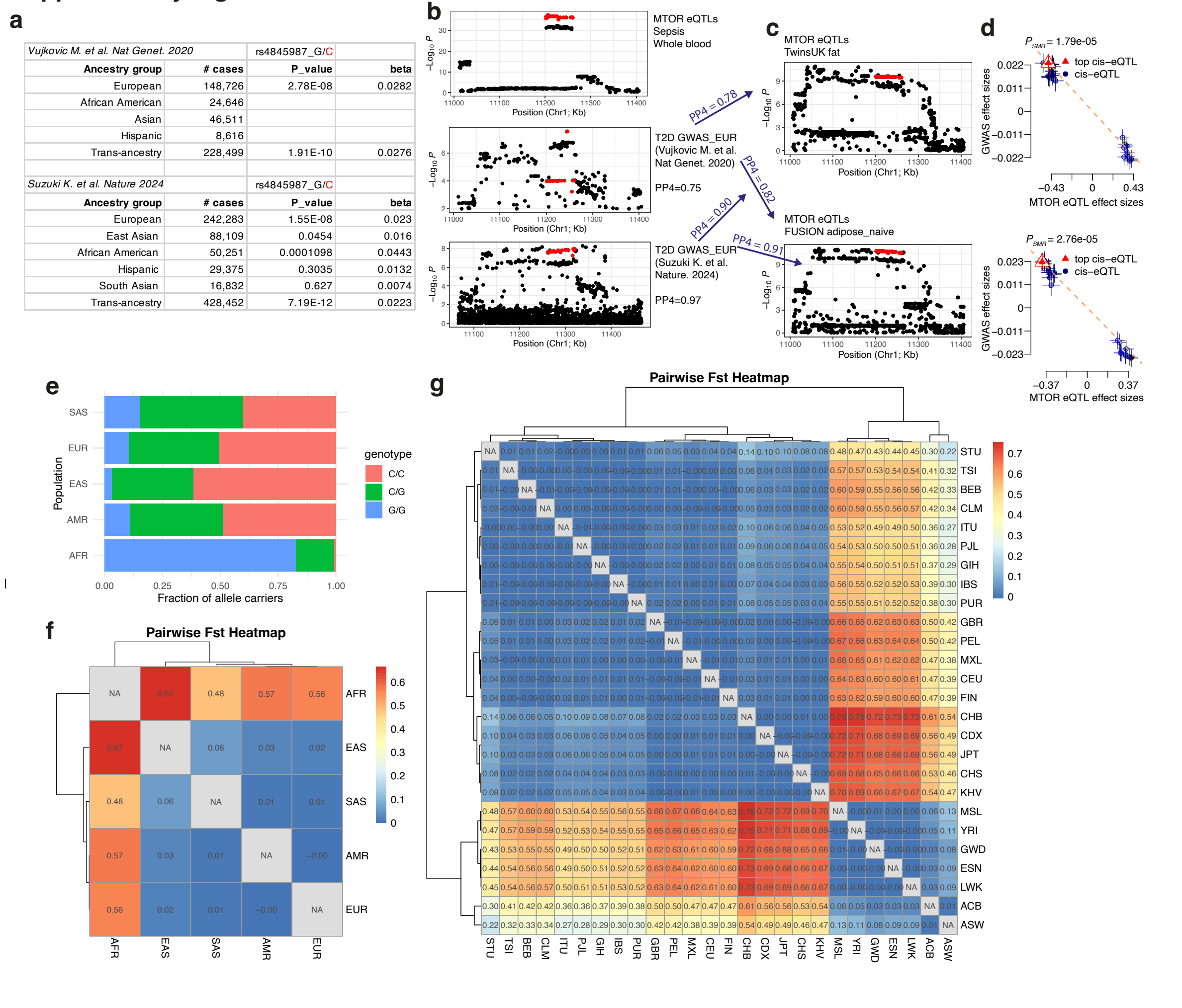

Vujkovic M. et al. Nat Genet. 2020		rs4845987_G/C	
**Ancestry group**	**# cases**	**P_value**	**beta**
European	148,726	2.78E-08	0.0282
African American	24,646		
Asian	46,511		
Hispanic	8,616		
Trans-ancestry	228,499	1.91E-10	0.0276
Suzuki K. et al. Nature 2024		rs4845987_G/C	
**Ancestry group**	**# cases**	**P_value**	**beta**
European	242,283	1.55E-08	0.023
East Asian	88,109	0.0454	0.016
African American	50,251	0.0001098	0.0443
Hispanic	29,375	0.3035	0.0132
South Asian	16,832	0.627	0.0074
Trans-ancestry	428,452	7.19E-12	0.0223

Supplementary Fig. 3. (**a**) T2D GWAS association results for rs4845987 across different ancestral groups. The effect allele C was highlighted in red. **(b-c)** Regional plots showing the colocalisation of the MTOR eQTL associations in sepsis whole blood, GWAS risk SNPs for T2D (b), and *MTOR* eQTLs identified in adipose tissue (c). (**d**) Dot plots showing the effect sizes of SNPs used for the SMR/HEIDI analysis from T2D GWAS [3] on the y axis, against eQTL data in adipose tissue on the x axis. Error bars represent the standard errors of the SNP effects. (**e**) Fraction of rs4845987 allele carriers across the five superpopulations of the 1000 Genomes Project. Genotype data was downloaded from http://ftp.1000genomes.ebi.ac.uk/vol1/ftp/data_collections/1000_genomes_project/release/. (**f-g**) Heatmaps of pairwise Fst for the 25 *MTOR* lead eQTLs (r^2>0.95) across 5 superpopulations (f), and 26 subpopulations (g) based on the 1000 Genomes Project data. Fst was calculated using Weir and Cockerham method as implemented in the R package hierfstat [4]. Abbreviations: AFR:African; AMR:American; EAS:East Asian; EUR:European; SAS:South Asian. ACB:African Caribbean; ASW:African Ancestry in Southwest US; BEB:Bengali; CDX:Dai Chinese; CEU:Utah residents (CEPH) with Northern and Western European; CHB:Han Chinese; CHS:Southern Han Chinese; CLM:Colombian; ESN:Esan in Nigeria; FIN:Finnish; GBR:British; GIH:Gujarati; GWD:Gambian Mandinka; IBS:Iberian; ITU:Telugu; JPT:Japanese; KHV:Kinh Vietnamese; LWK:Luhya; MSL:Mende; MXL:Mexican; PEL:Peruvian; PJL:Punjabi; PUR:Puerto Rican; STU:Tamil; TSI: Toscani; YRI: Yoruba.

Supplementary Figure 4

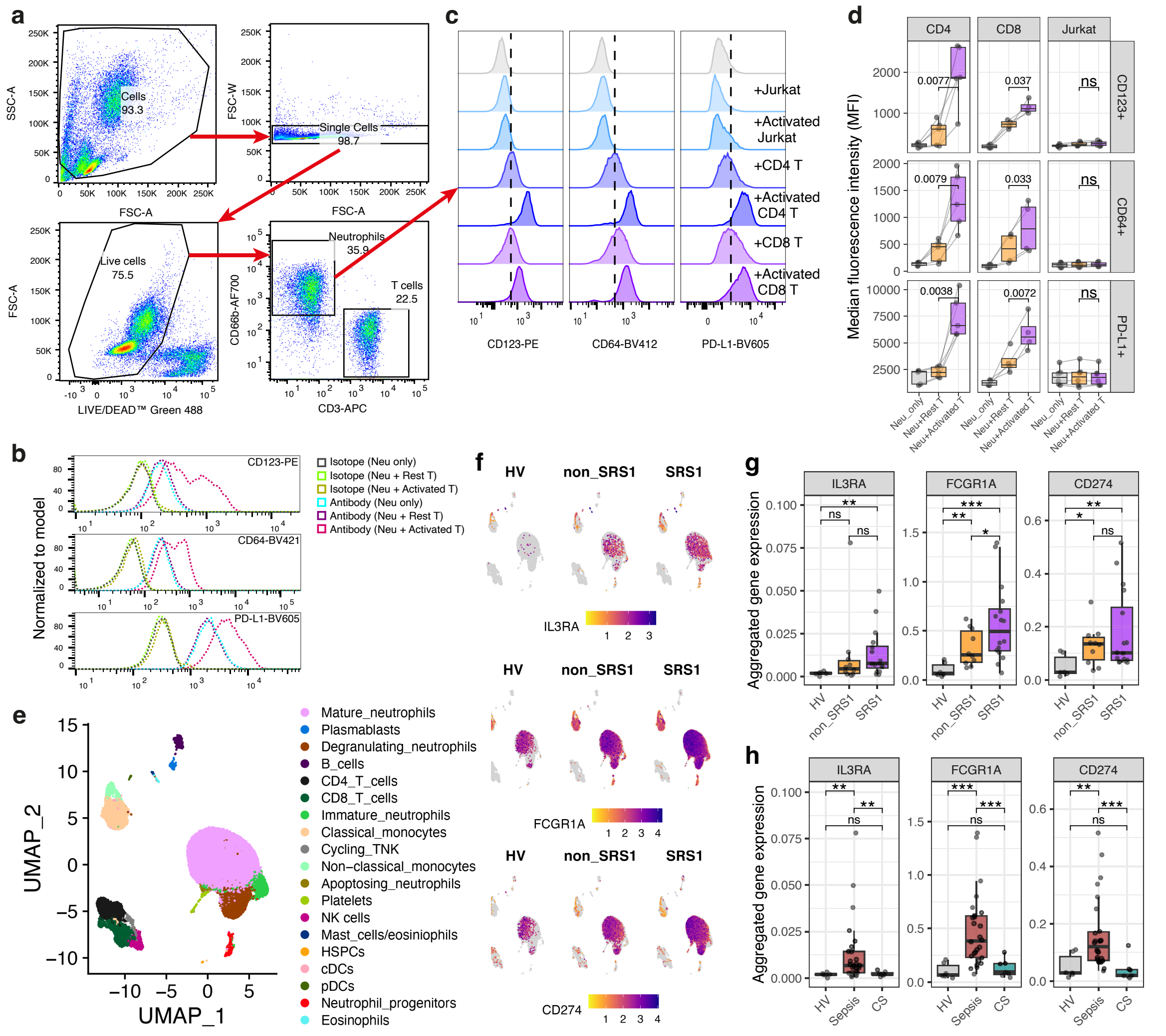

Supplementary Fig. 4. (**a**) Flow cytometry gating strategy for Neu-to-T cell co-culture. Viable neutrophils were gated using LIVE/DEAD™ Fixable Green Dead Cell Stain Kit (ThermoFisher) (see Methods) (**b**) Flow cytometry histograms showing surface marker expression on sepsis neutrophils after 2 days of co-culture with or without allogeneic T cells. The matched isotope controls for CD123-PE (BD Biosciences, #558595), CD64-BV421 (Biolegend, #400157) and PD-L1-BV605 (Biolegend, #400349) antibodies were used for this analysis.(**c**) Flow cytometry panels showing the upregulation of CD123, CD64 and PD-L1 expression on sepsis neutrophils co-cultured with allogeneic Jurkat, primary CD4+ or CD8+ T cell with or w/o anti-CD3/CD28 Dynabeads. (**d**) Bar plot showing the flow cytometry quantifications for CD64, CD123 and PD-L1 levels in sepsis neutrophils in the presence of Jurkat, or primary resting T cells with or w/o anti-CD3/CD28 Dynabeads. P values were calculated by paired t-test. (**e-f**) UMAP projection of the *Kwok et al.* whole blood sepsis scRNA-seq dataset [5] coloured according to broad cell lineages (e), and for the expression of *IL3RA* (encoding CD123), *FCGR1A* (CD64) and *CD274* (PD-L1) in healthy volunteers (HV), non-SRS1 and SRS1 sepsis patients (f). Data was visualised using R packages *Seurat* and *scCustomize*. (**g-h**) Boxplots showing the transcript levels of IL3RA, FCGR1A and CD274 in neutrophils from healthy volunteers (HV; n=6), non-SRS1 (n=10) and SRS1 (n=16) sepsis patients, and patients after cardiac surgery (CS; n=7). Data are derived from whole-blood sepsis scRNA-seq[5]. Normalised expression values were averaged across cells per sample (sample-level aggregates). Group comparisons were tested using two-tailed t-tests. Source data for panels **d** are provided as a Source Data file.

Supplementary Figure 5

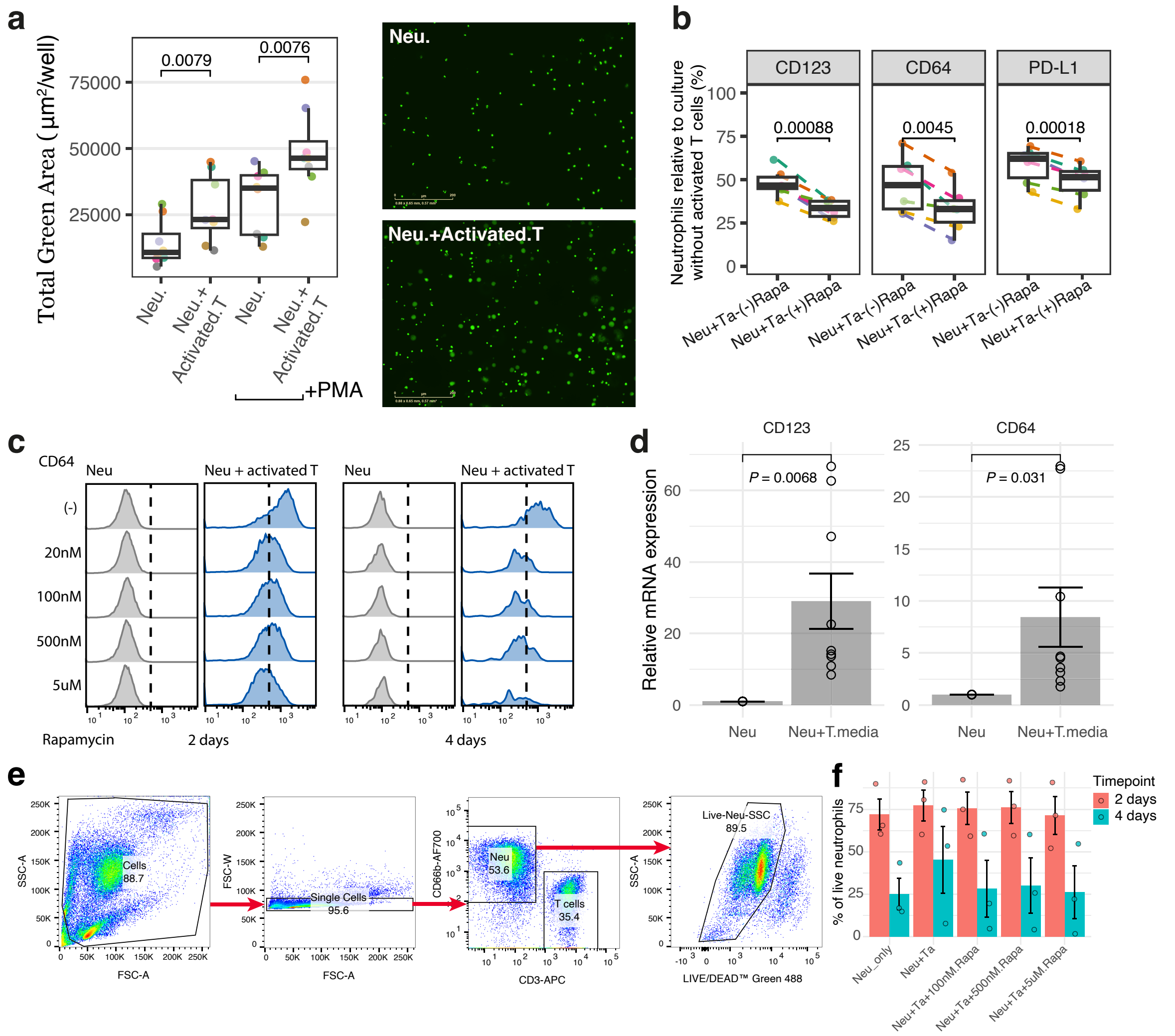

Supplementary Fig. 5. (**a**) Quantification of NETosis using Incucyte S3 live cell analysis instrument and Cytotox green dye with or without PMA treatment for 4-6 hours (left panel), and the representative images (right panels) showing the enhanced NETosis on neutrophils when co-cultured with activated T cells. For quantification, the green objects <300 µm^2 (apoptotic cells) were filtered out (n=8). (**b**) Box plots showing the expression of markers on sepsis neutrophils in the presence of activated T cells with or w/o rapamycin pre-treatment (100nM for 2 days) (n=6). *P* value was calculated by two-tailed paired t-test. (**c**) Flow cytometry histograms for the CD64 expression on sepsis neutrophil co-cultured with activated T cells in the presence of rapamycin with different concentrations for 2 days (left panels) or 4 days (right panels). (**d**) Bar plots of gene expression measured using qRT-PCR normalized to β-Actin ($2^{-\Delta\Delta Ct}$) in sepsis neutrophils in the presence of conditioned media from T cells treated with anti-CD3/CD28 beads for 2days. Error bars represent SEM of 9 independent replicates. P value was calculated by two-tailed t-test. (**e**) Gating strategy for quantifying viable neutrophils co-cultured with activated T cells. (**f**) Bar plots show the percentage of viable sepsis neutrophils after 2 or 4 days of co-culture with increasing concentrations of rapamycin. Error bars represent the mean ± SEM from 3 independent replicates. Each dot represents an individual patient sample across conditions. Source data for panels **a, b, d, f** are provided as a Source Data file.

Supplementary Figure 6

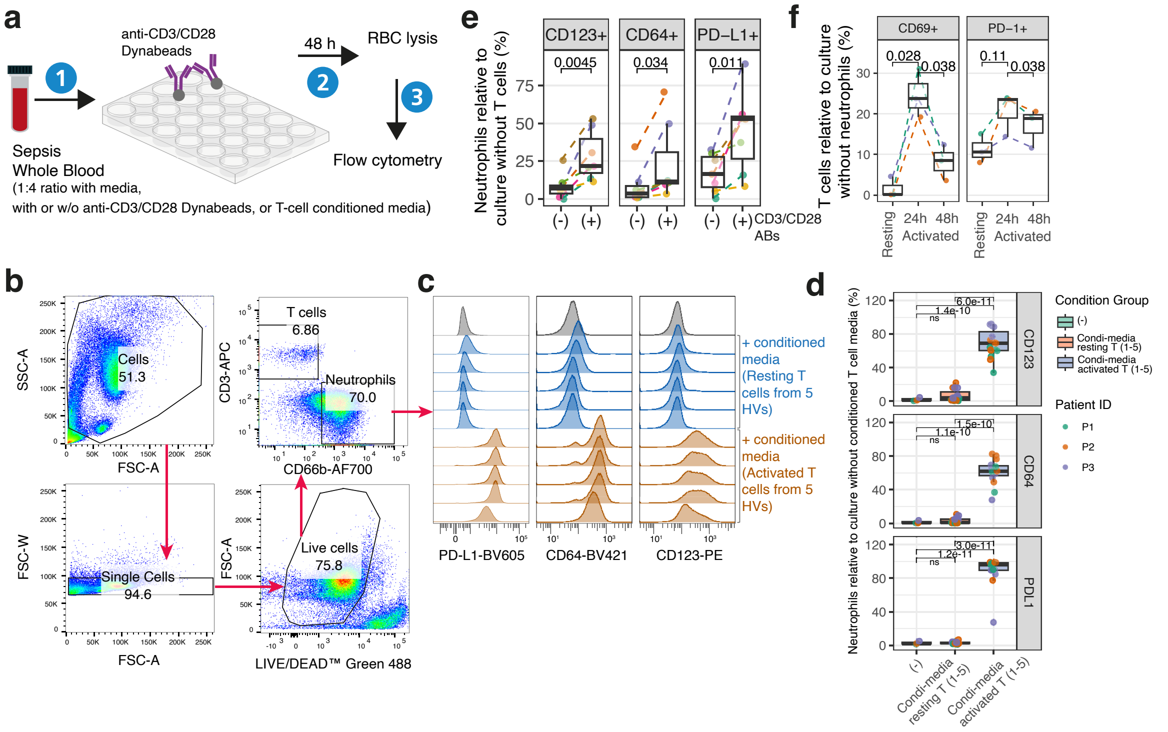

Supplementary Fig. 6. (**a**) Schematic of *ex vivo* co-culture of sepsis whole blood collected by a BD Vacutainer EDTA tube with anti-CD3/CD28 Dynabeads or T cell conditioned media. (**b**) Flow cytometry gating strategy for viable neutrophils and T cells from whole blood using surface markers CD66b, CD3 and the LIVE/DEAD™ Fixable Green Dead Cell Stain Kit (ThermoFisher) (see **Methods**). (**c**) Flow cytometry histograms showing surface marker expression on viable CD66b+ sepsis neutrophils of whole blood cultured either alone (grey) or with conditioned media from resting T cells (blue) or activated T cells (orange) at a 1:4 ratio for 2 days. T cells derived from 5 different healthy donors were cultured with (activated) or without (resting) anti-CD3/CD28 Dynabeads at 1 x 10^6 cells/ml. T cell conditioned media (supernatant) was harvested in 2 days. (**d**) Quantification of expression markers on CD66b+ neutrophils in whole blood from sepsis patients (n=3) cultured with conditioned T cell media derived from 5 healthy donors. P value was calculated by two-tailed t-test. ns: not significant. (**e**) Relative marker expression on CD66b+ neutrophils in whole blood from sepsis patients (n=7) with or without anti-CD3/CD28 T cell activation for 48 hours. (**f**) Relative expression of CD69 and PD-1 on CD3+ T cells in sepsis whole blood (n=3) following anti-CD3/CD28 activation for 24 or 48 hours. P values were calculated using a two-tailed paired t-test. Source data for panels **d, e, f** are provided as a Source Data file.

Supplementary Figure 7

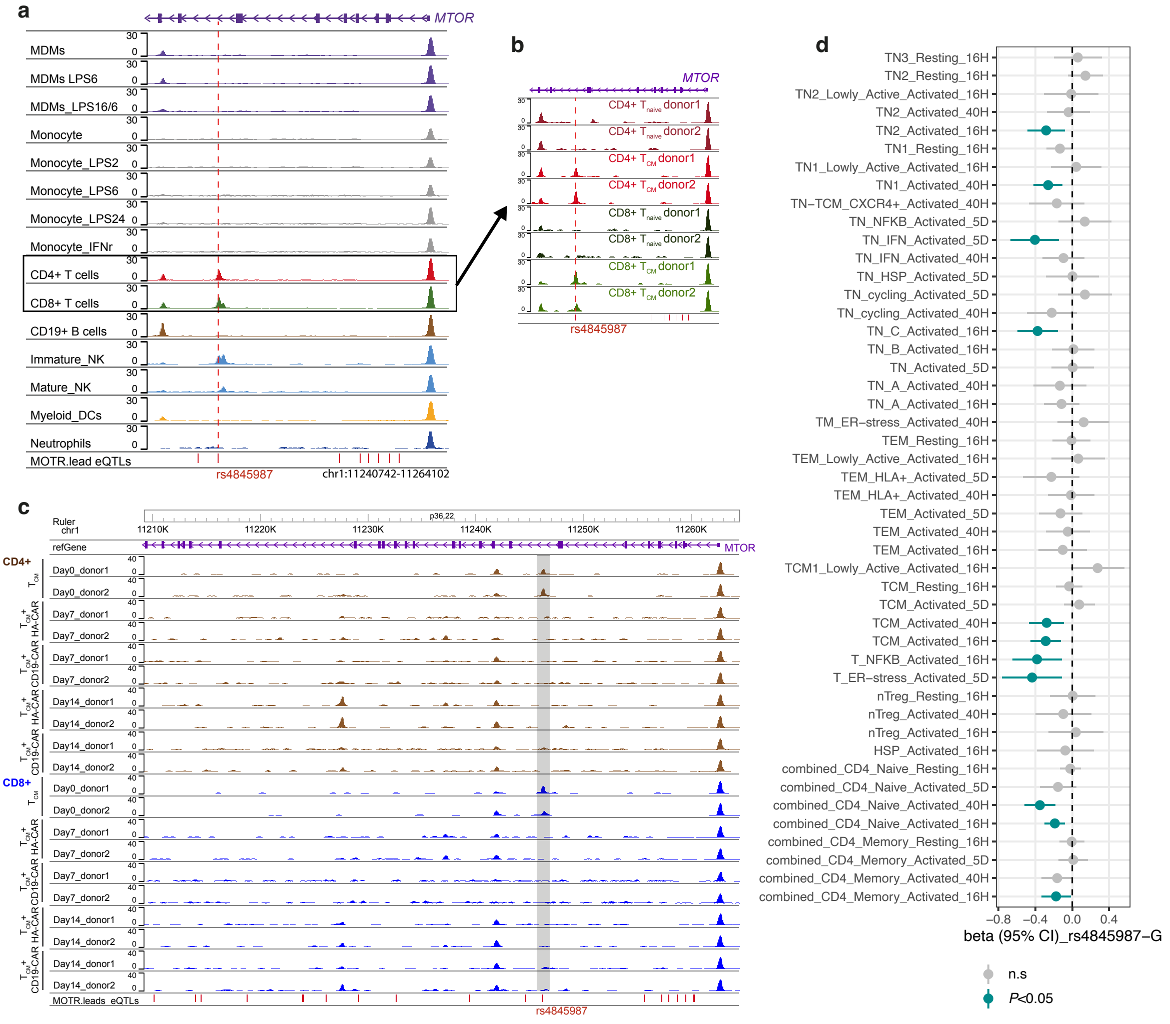

Supplementary Fig. 7. (a) Genome browser tracks showing a *MTOR* eQTL (in red dashed line) resides in ATACseq peaks of CD4+ and CD8+ T cells. ATAC-seq raw data in different immune cells listed in **Supplementary Table 6**, were uniformly processed (see **Method**) and the average sequencing depth for each dataset is shown on the y axis. (**b-c**) ATAC-seq tracks showing the variant-containing enhancer of the *MTOR* locus in CD4+ and CD8+ central memory T (T_{CM}) cells derived from two different healthy donors (b), followed by GD2-targeting (HA-28ζ) and CD19-targeting (CD19-28ζ) CAR T cell production with anti-CD3/CD28 Dynabeads (c). Sequencing data were downloaded from GSE168882[6]. (**d**) eQTL associations for *MTOR* across CD4+ T cell subsets based on a sc-RNAseq dataset[7]. Cells were activated with anti-CD3/CD28 Dynabeads for 16 hours (16H), 40 hours (40H) or 5 days (5D). Association data were retrieved from eQTL Catalogue[8].

Supplementary Figure 8

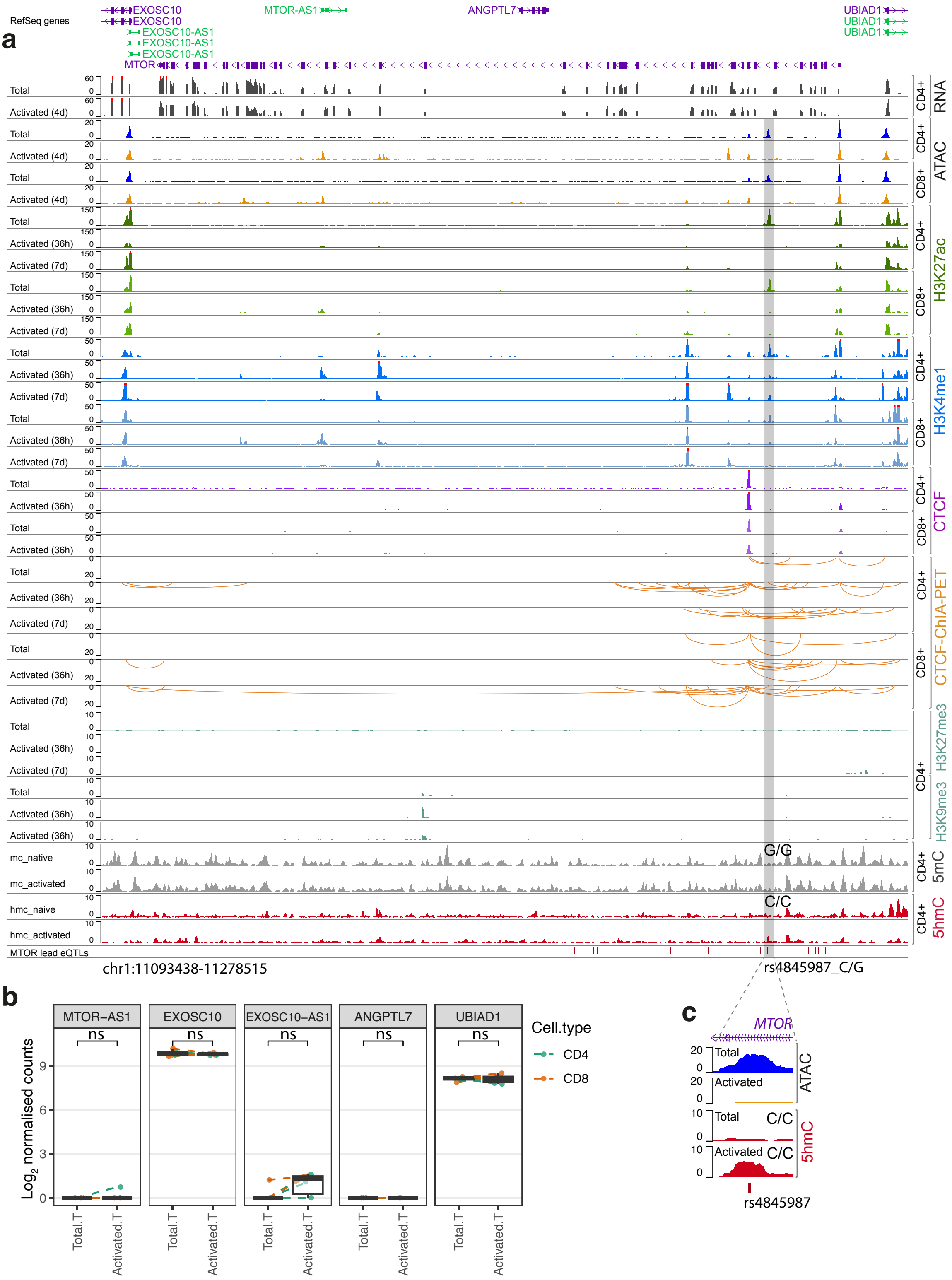

Supplementary Fig. 8. (**a**) Genome browser tracks showing the variant-containing enhancer (highlighted in grey) in the *MTOR* locus. Processed signal files for histone modifications and CTCF ChIP-seq and CTCF ChIA-PET looping data were downloaded from the Encyclopedia of DNA Elements (ENCODE) project[9] (**Supplementary Table 7**). Only interaction loops with PET count score more than or equal to 5 are shown. (**b**) Box plot showing the expression changes of the enhancer-surrounding gene transcripts including *MTOR-AS1, EXOSC10, EXOSC10-AS1, ANGPTL7* and *UBIAD1* in T cells upon activation (n=6 from 3 healthy donors). (**c**) MeDIP-seq data for 5hmC modification in resting and

activated CD4+ T cells (anti-CD3/CD28 for 2-3days) (GSE74850)[10] were analysed as described in the Methods section. Sample genotype was determined using the aligned sequence reads.

Supplementary Figure 9

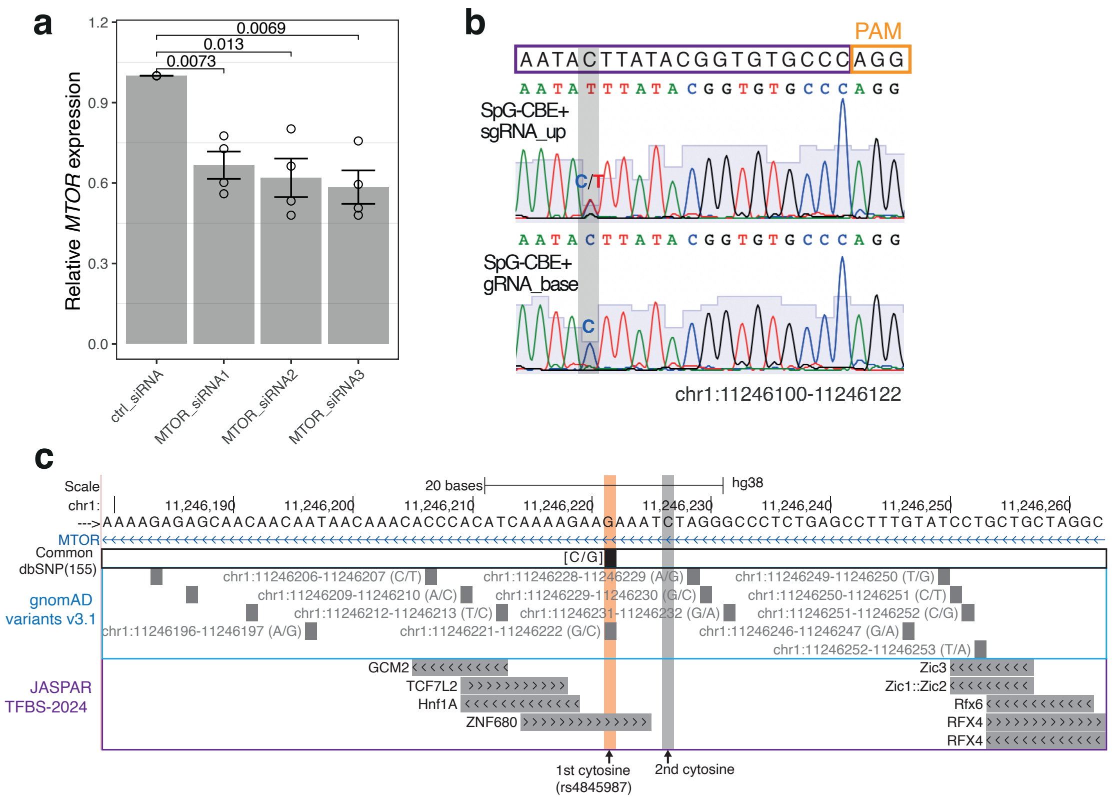

Supplementary Fig. 9. (**a**) Bar plot showing relative MTOR transcript levels ($2^{-\Delta\Delta Ct}$) in activated T cells electroporated with either non-targeting control siRNA or three distinct siRNAs targeting MTOR mRNA. Primary T cells were pre-activated with anti-CD3/CD28 beads for 2 days before electroporation. The synthesized siRNA (~300nM; sequences are listed in Supplementary Table 8) were then delivered using the P3 Primary Cell 4D-Nucleofector Kit (Lonza, #V4XP-3032). Cells were harvested 6 days post-electroporation for RNA extraction and qRT-PCR analyses. P values were calculated using a two-tailed t-test on 4 independent replicates from 2 healthy donors. (**b**) Sanger sequencing chromatograms of the sgRNA-up locus in cells electroporated with SpG-TadCBE6b mRNA and either sgRNA-up (upper panel) or sgRNA-base (lower panel). (**c**) UCSC Genome Browser tracks showing the *MTOR* eQTL (orange) and the 2nd cytosine with the bystander edit (grey), with annotation tracks including common SNPs (dbSNP155), variants from 76,156 whole genomes (gnomAD v3.1), and predicted transcription factor binding sites from the JASPAR 2024 database. Source data for panel **a** are provided as a Source Data file.

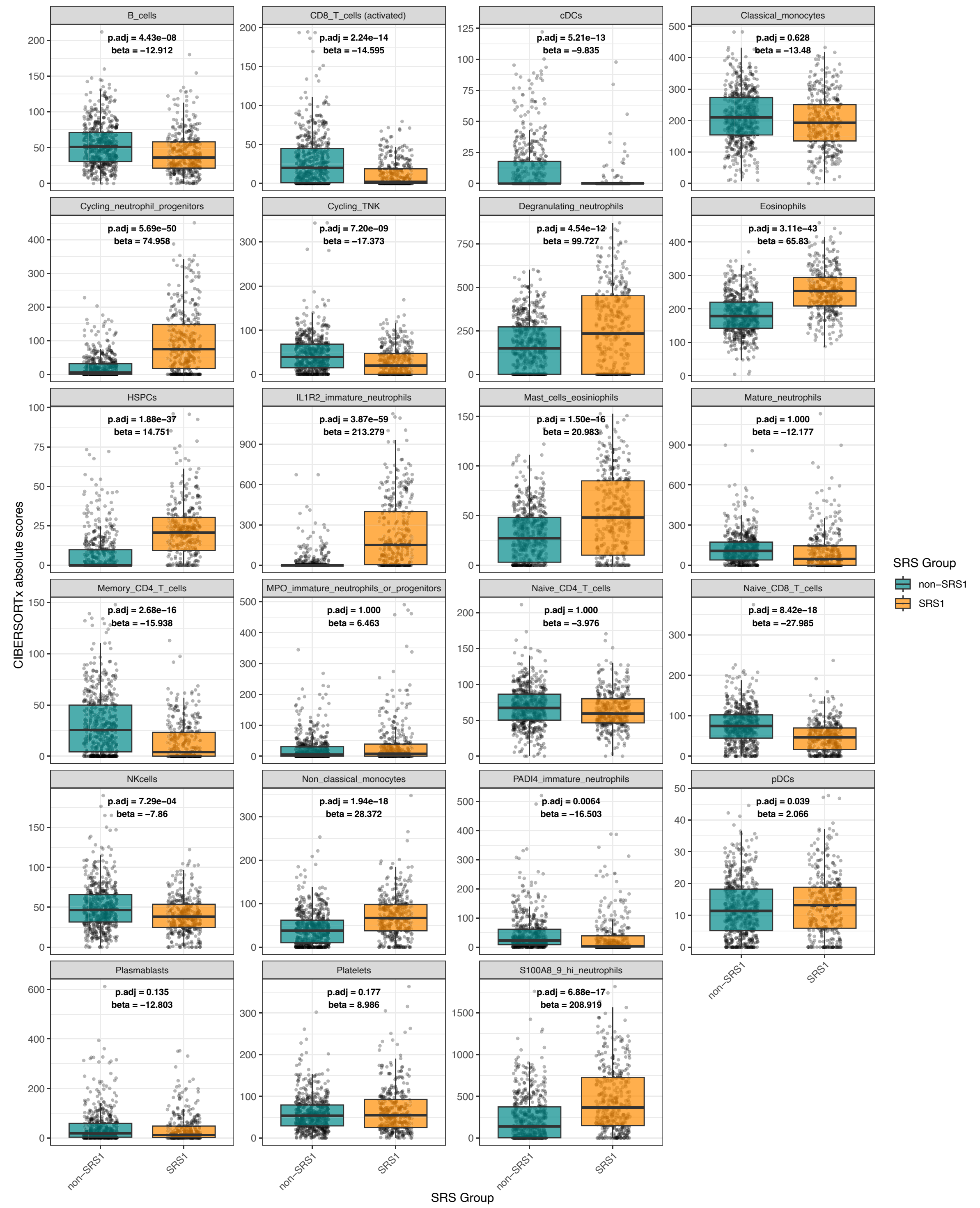

Supplementary Fig. 10. Box plots showing CIBERSORTx absolute scores across 23 deconvoluted cell subsets (see **Methods**). *P* values comparing SRS1 and non-SRS1 groups were calculated using a linear mixed model accounting for donor variability and corrected for multiple testing using Bonferroni.

References

1. COVID-19 Multi-omics Blood ATlas Consortium (2022). A blood atlas of COVID-19 defines hallmarks of disease severity and specificity. Cell *185*, 916-938 e958. 10.1016/j.cell.2022.01.012.

2. Torrance, H.D., Zhang, P., Longbottom, E.R., Mi, Y., Whalley, J.P., Allcock, A., Kwok, A.J., Cano-Gamez, E., Geoghegan, C.G., Burnham, K.L., et al. (2023). A Transcriptomic Approach to Understand Patient Susceptibility to Pneumonia After Abdominal Surgery. Ann Surg. 10.1097/SLA.0000000000006050.
3. Suzuki, K., Hatzikotoulas, K., Southam, L., Taylor, H.J., Yin, X., Lorenz, K.M., Mandla, R., Huerta-Chagoya, A., Melloni, G.E.M., Kanoni, S., et al. (2024). Genetic drivers of heterogeneity in type 2 diabetes pathophysiology. Nature *627*, 347-357. 10.1038/s41586-024-07019-6.
4. Goudet, J. (2005). HIERFSTAT, a package for R to compute and test hierarchical-statistics. Mol Ecol Notes *5*, 184-186. 10.1111/j.1471-8286.2004.00828.x.
5. Kwok, A.J., Allcock, A., Ferreira, R.C., Cano-Gamez, E., Smee, M., Burnham, K.L., Zurke, Y.X., Emergency Medicine Research, O., McKechnie, S., Mentzer, A.J., et al. (2023). Neutrophils and emergency granulopoiesis drive immune suppression and an extreme response endotype during sepsis. Nature immunology *24*, 767-779. 10.1038/s41590-023-01490-5.
6. Gennert, D.G., Lynn, R.C., Granja, J.M., Weber, E.W., Mumbach, M.R., Zhao, Y., Duren, Z., Sotillo, E., Greenleaf, W.J., Wong, W.H., et al. (2021). Dynamic chromatin regulatory landscape of human CAR T cell exhaustion. Proceedings of the National Academy of Sciences of the United States of America *118*. 10.1073/pnas.2104758118.
7. Soskic, B., Cano-Gamez, E., Smyth, D.J., Ambridge, K., Ke, Z., Matte, J.C., Bossini-Castillo, L., Kaplanis, J., Ramirez-Navarro, L., Lorenc, A., et al. (2022). Immune disease risk variants regulate gene expression dynamics during CD4(+) T cell activation. Nature genetics *54*, 817-826. 10.1038/s41588-022-01066-3.
8. Kerimov, N., Hayhurst, J.D., Peikova, K., Manning, J.R., Walter, P., Kolberg, L., Samovica, M., Sakthivel, M.P., Kuzmin, I., Trevanion, S.J., et al. (2021). A compendium of uniformly processed human gene expression and splicing quantitative trait loci. Nature genetics *53*, 1290-1299. 10.1038/s41588-021-00924-w.
9. Consortium, E.P. (2012). An integrated encyclopedia of DNA elements in the human genome. Nature *489*, 57-74. 10.1038/nature11247.
10. Marina, R.J., Sturgill, D., Bailly, M.A., Thenoz, M., Varma, G., Prigge, M.F., Nanan, K.K., Shukla, S., Haque, N., and Oberdoerffer, S. (2016). TET-catalyzed oxidation of intragenic 5-methylcytosine regulates CTCF-dependent alternative splicing. The EMBO journal *35*, 335-355. 10.15252/embj.201593235.
