# Supplementary material for: Context-specific regulatory genetic variation in MTOR dampens neutrophil-T cell crosstalk in pneumonia-associated sepsis
Source: Nat Commun. 2026 Feb 25;17:3201. doi: 10.1038/s41467-026-69919-7 (PMC13057173; doi:10.1038/s41467-026-69919-7)
Supplement: Supplementary file 2 — Description of Additional Supplementary Files [file 41467_2026_69919_MOESM2_ESM.pdf]

Title: Supplementary Table 1

Description: Genome-wide sepsis eQTLs showing interaction with neutrophil-to-lymphocyte ratio (NLR).

Title: Supplementary Table 2

Description: Colocalisation results between the MTOR sepsis eQTL and eQTLs across different cell types and treatment conditions.

Title: Supplementary Table 3

Description: GWAS traits significantly associated with rs4845987 or linked variants in strong linkage disequilibrium.

Title: Supplementary Table 4

Description: Summary data-based Mendelian randomisation results linking MTOR expression to type 2 diabetes risk across cell types.

Title: Supplementary Table 5

Description: Differentially expressed cytokine genes in activated $CD4^+$ and $CD8^+$ T cells following anti-CD3/CD28 stimulation.

Title: Supplementary Table 6

Description: Epigenomic datasets used to assess chromatin accessibility and regulatory features at the MTOR locus.

Title: Supplementary Table 7

Description: ENCODE histone modification and CTCF ChIP-seq datasets analysed at the MTOR locus.

Title: Supplementary Table 8

Description: sgRNA, primer and oligonucleotide sequences used for qRT-PCR, Sanger sequencing, RNAi, CRISPR-based epigenetic editing and base-editing experiments.
