# Supplementary material for: Context-specific regulatory genetic variation in MTOR dampens neutrophil-T cell crosstalk in pneumonia-associated sepsis
Source: Nat Commun. 2026 Feb 25;17:3201. doi: 10.1038/s41467-026-69919-7 (PMC13057173; doi:10.1038/s41467-026-69919-7)
Supplement: Supplementary file 4 — Reporting Summary [file 41467_2026_69919_MOESM4_ESM.pdf]

nature portfolio

Corresponding author(s): Ping Zhang; Julian C. Knight

Last updated by author(s): 5 November 2025

# Reporting Summary

Nature Portfolio wishes to improve the reproducibility of the work that we publish. This form provides structure for consistency and transparency in reporting. For further information on Nature Portfolio policies, see our Editorial Policies and the Editorial Policy Checklist.

## Statistics

For all statistical analyses, confirm that the following items are present in the figure legend, table legend, main text, or Methods section.

| n/a | Confirmed | |
|---|---|---|
| [ ] | [x] | The exact sample size (*n*) for each experimental group/condition, given as a discrete number and unit of measurement |
| [ ] | [x] | A statement on whether measurements were taken from distinct samples or whether the same sample was measured repeatedly |
| [ ] | [x] | The statistical test(s) used AND whether they are one- or two-sided<br>*Only common tests should be described solely by name; describe more complex techniques in the Methods section.* |
| [ ] | [x] | A description of all covariates tested |
| [ ] | [x] | A description of any assumptions or corrections, such as tests of normality and adjustment for multiple comparisons |
| [ ] | [x] | A full description of the statistical parameters including central tendency (e.g. means) or other basic estimates (e.g. regression coefficient) AND variation (e.g. standard deviation) or associated estimates of uncertainty (e.g. confidence intervals) |
| [ ] | [x] | For null hypothesis testing, the test statistic (e.g. *F*, *t*, *r*) with confidence intervals, effect sizes, degrees of freedom and *P* value noted<br>*Give P values as exact values whenever suitable.* |
| [ ] | [x] | For Bayesian analysis, information on the choice of priors and Markov chain Monte Carlo settings |
| [x] | [ ] | For hierarchical and complex designs, identification of the appropriate level for tests and full reporting of outcomes |
| [ ] | [x] | Estimates of effect sizes (e.g. Cohen's *d*, Pearson's *r*), indicating how they were calculated |

*Our web collection on statistics for biologists contains articles on many of the points above.*

## Software and code

Policy information about availability of computer code

**Data collection**: No special software was used for data collection.

**Data analysis**:

| Tool/software | Version | Context |
|---|---|---|
| R | 4.2.1 | Statistical analyses and modeling |
| DESeq2 (R package) | 1.36.1 | Differential expression analysis |
| lmerTest (R package) | 3.1.3 | Linear mixed model for eQTL interaction analysis |
| coloc (R package) | 5.1.0.1 | Colocalisation analysis for eQTLs |
| hierfstat (R package) | 0.5.11 | Fixation index (Fst) calculation for population genetics |
| Seurat (R package) | 4.3.0.1 | scRNA-seq data visualisation and analysis |
| scCustomize (R package) | 1.1.3 | scRNA-seq data visualisation |
| SepstratifieR | 1 | Sepsis response signature (SRS) estimation |
| SMR tool | 1.3.1 | Mendelian randomisation using eQTL and GWAS data |
| featureCounts | 1.6.4 | Generation of RNA-seq count matrices |
| HISAT2 | 2.1.0 | Alignment of RNA-seq reads |
| Trim Galore | 0.6.2 | Read trimming for sequencing data |
| Bowtie2 | 2.2.5 | Alignment of ATAC-seq reads |
| Picard | 2.0.1 / 2.21.1 | Sequence data QC and duplicate removal |
| Samtools | 1.9 | BAM file manipulation and filtering |
| MACS2 | 2.1.0 | Peak calling for ATAC-seq and hMeDIP-seq |

| | | |
|---|---|---|
| deepTools 3.3.1 | Generation of normalised bigwig coverage tracks | |
| BWA-mem | 0.7.12 | Alignment for hMeDIP-seq data |
| FlowJo | 10.10 | Flow cytometry data analysis |

All code used for data processing and analysis in this study is publicly available on GitHub (https://github.com/jknightlab/MTOR-Genetics-Project).

For manuscripts utilizing custom algorithms or software that are central to the research but not yet described in published literature, software must be made available to editors and reviewers. We strongly encourage code deposition in a community repository (e.g. GitHub). See the Nature Portfolio guidelines for submitting code & software for further information.

## Data

Policy information about availability of data

All manuscripts must include a data availability statement. This statement should provide the following information, where applicable:

- Accession codes, unique identifiers, or web links for publicly available datasets
- A description of any restrictions on data availability
- For clinical datasets or third party data, please ensure that the statement adheres to our policy

GAinS gene expression and genotyping data were previously deposited at the European Genome-phenome Archive (EGA), under accession number EGAD00001008730 and EGAD00001015369. RNA-seq and ATAC-seq raw FASTQ files for CD4+ and CD8+ T cells are available under accession number EGAS50000000894 (https://ega-archive.org/studies/EGAS50000000894). Processed data, including raw and normalised counts and bigWig files for genome-wide signal data can be accessed on Zenodo (https://zenodo.org/uploads/14907264). The raw ATAC-seq data for primary immune cells were obtained from GSE172116 (macrophages), EGAS00001007362 (monocytes), GSE150018 (neutrophils), GSE118189 (NK and dendritic cells) and GSE168882 (CAR T cells). RNA-seq data in rapamycin-treated CD4+ T cells were obtained from GSE129829. MeDIP-Seq for 5hmC in CD4+ T cells were obtained from GSE74850. The processed histone modification and CTCF ChIP-seq results were downloaded from the ENCODE project.

## Research involving human participants, their data, or biological material

Policy information about studies with human participants or human data. See also policy information about sex, gender (identity/presentation), and sexual orientation and race, ethnicity and racism.

| | |
|---|---|
| Reporting on sex and gender | Sex was determined by self-reporting. Analyses were adjusted for sex where applicable; sex-specific analyses were not performed. The GAinS cohort included 1,168 patients with genotype data available (54% male; aged 18-92 years), predominantly of European ancestry (96%). The SI-CAP cohort used in this study comprised 102 European patients (52% male; aged 19-98 years) who were genotyped for the MTOR SNP rs4845987. From the UK Biobank bacterial pneumonia cohort, self-reported sex in instance 0 of field 31 was used to define males (n=696) and females (n=429). |
| Reporting on race, ethnicity, or other socially relevant groupings | Ethnicity was determined genetically in cohorts with available genotype data. Analyses were restricted to individuals of European ancestry, and the first seven genotype principal components were included as covariates where applicable to account for potential effects of ethnicity. |
| Population characteristics | The GAinS cohort included 1,168 patients with genotype data available (54% men; aged 18-92 years), predominantly of European ancestry (96%). The SI-CAP cohort used in this study comprised 102 self-reported European patients (52% male; aged 19-98 years) who were genotyped for the MTOR SNP rs4845987. From UK Biobank cohort, patients with a genetically determined ancestry of White British Subset (WBS) were included. Additional summary-level data on population characteristics are provided in the original manuscripts cited in the paper. |
| Recruitment | Patients with sepsis were recruited from the Genomic Advances in Sepsis (GAinS) and Sepsis Immunomics (SI) studies. The inclusion and exclusion criteria for both cohorts have been described previously (PMID: 28036233, 37095375). The UK Biobank recruited approximately half a million men and women aged 40-69 years attended one of 22 UKB assessment centres located throughout England, Scotland and Wales between 2006 and 2010 as described previously (PMID: 25826379). |
| Ethics oversight | Peripheral blood samples were obtained from healthy volunteers following informed consent (Oxfordshire Research Ethics Committee approval REC reference 06/Q1605/55); and from sepsis patients in the Sepsis Immunomics (SI) study (South Central Oxford REC C, reference:19/SC/0296) and UK GAinS (REC approvals 05/MRE00/38, 08/H0505/78, and 06/Q1605/55) with ethics approval granted nationally and locally, and informed consent obtained from all patients or their legal representative. UK Biobank has obtained ethics approval from the North West Multi-centre Research Ethics Committee (approval number: 11/NW/0382) and had obtained informed consent from all participants. |

Note that full information on the approval of the study protocol must also be provided in the manuscript.

# Field-specific reporting

Please select the one below that is the best fit for your research. If you are not sure, read the appropriate sections before making your selection.

[x] Life sciences  [ ] Behavioural & social sciences  [ ] Ecological, evolutionary & environmental sciences

For a reference copy of the document with all sections, see nature.com/documents/nr-reporting-summary-flat.pdf

# Life sciences study design

All studies must disclose on these points even when the disclosure is negative.

| | |
|---|---|
| Sample size | The eQTL interaction analysis were performed using 823 RNA-seq samples derived from 638 sepsis patients. The survival analysis for 28-day mortality was conducted in 737 individuals of European ancestry of sepsis due to pneumonia, and replicated in 102 individuals of the SI-CAP patients with hospitalisation dates up to 04-12-2024, and 1,125 individuals of the UKB cohort.<br>The number of datasets to be used was not predetermined and no sample size calculation was made prior to this study. |
| Data exclusions | Patients with non-European ancestry were excluded from the survival analysis. |
| Replication | Survival analysis for 28-day mortality was performed in the GAinS cohort and replicated in the SI-CAP and UKB cohorts.<br>At least four independent biological replicates derived from different donors were used for the functional experiments as indicated in the figure legends. |
| Randomization | There was no a priori allocation of participants to separate groups. Stratified analyses were performed based on the presence or absence of defined phenotypes. |
| Blinding | Transcriptional profiling, SRS assignment and genotyping were performed with blinding to clinical metadata. |

# Behavioural & social sciences study design

All studies must disclose on these points even when the disclosure is negative.

| | |
|---|---|
| Study description | *Briefly describe the study type including whether data are quantitative, qualitative, or mixed-methods (e.g. qualitative cross-sectional, quantitative experimental, mixed-methods case study).* |
| Research sample | *State the research sample (e.g. Harvard university undergraduates, villagers in rural India) and provide relevant demographic information (e.g. age, sex) and indicate whether the sample is representative. Provide a rationale for the study sample chosen. For studies involving existing datasets, please describe the dataset and source.* |
| Sampling strategy | *Describe the sampling procedure (e.g. random, snowball, stratified, convenience). Describe the statistical methods that were used to predetermine sample size OR if no sample-size calculation was performed, describe how sample sizes were chosen and provide a rationale for why these sample sizes are sufficient. For qualitative data, please indicate whether data saturation was considered, and what criteria were used to decide that no further sampling was needed.* |
| Data collection | *Provide details about the data collection procedure, including the instruments or devices used to record the data (e.g. pen and paper, computer, eye tracker, video or audio equipment) whether anyone was present besides the participant(s) and the researcher, and whether the researcher was blind to experimental condition and/or the study hypothesis during data collection.* |
| Timing | *Indicate the start and stop dates of data collection. If there is a gap between collection periods, state the dates for each sample cohort.* |
| Data exclusions | *If no data were excluded from the analyses, state so OR if data were excluded, provide the exact number of exclusions and the rationale behind them, indicating whether exclusion criteria were pre-established.* |
| Non-participation | *State how many participants dropped out/declined participation and the reason(s) given OR provide response rate OR state that no participants dropped out/declined participation.* |
| Randomization | *If participants were not allocated into experimental groups, state so OR describe how participants were allocated to groups, and if allocation was not random, describe how covariates were controlled.* |

# Ecological, evolutionary & environmental sciences study design

All studies must disclose on these points even when the disclosure is negative.

| | |
|---|---|
| Study description | *Briefly describe the study. For quantitative data include treatment factors and interactions, design structure (e.g. factorial, nested, hierarchical), nature and number of experimental units and replicates.* |
| Research sample | *Describe the research sample (e.g. a group of tagged Passer domesticus, all Stenocereus thurberi within Organ Pipe Cactus National Monument), and provide a rationale for the sample choice. When relevant, describe the organism taxa, source, sex, age range and any manipulations. State what population the sample is meant to represent when applicable. For studies involving existing datasets, describe the data and its source.* |
| Sampling strategy | *Note the sampling procedure. Describe the statistical methods that were used to predetermine sample size OR if no sample-size calculation was performed, describe how sample sizes were chosen and provide a rationale for why these sample sizes are sufficient.* |
| Data collection | *Describe the data collection procedure, including who recorded the data and how.* |
| Timing and spatial scale | *Indicate the start and stop dates of data collection, noting the frequency and periodicity of sampling and providing a rationale for* |

| | |
|---|---|
| | *these choices. If there is a gap between collection periods, state the dates for each sample cohort. Specify the spatial scale from which the data are taken* |
| Data exclusions | *If no data were excluded from the analyses, state so OR if data were excluded, describe the exclusions and the rationale behind them, indicating whether exclusion criteria were pre-established.* |
| Reproducibility | *Describe the measures taken to verify the reproducibility of experimental findings. For each experiment, note whether any attempts to repeat the experiment failed OR state that all attempts to repeat the experiment were successful.* |
| Randomization | *Describe how samples/organisms/participants were allocated into groups. If allocation was not random, describe how covariates were controlled. If this is not relevant to your study, explain why.* |
| Blinding | *Describe the extent of blinding used during data acquisition and analysis. If blinding was not possible, describe why OR explain why blinding was not relevant to your study.* |

Did the study involve field work? ☐ Yes ☐ No

## Field work, collection and transport

| | |
|---|---|
| Field conditions | *Describe the study conditions for field work, providing relevant parameters (e.g. temperature, rainfall).* |
| Location | *State the location of the sampling or experiment, providing relevant parameters (e.g. latitude and longitude, elevation, water depth).* |
| Access & import/export | *Describe the efforts you have made to access habitats and to collect and import/export your samples in a responsible manner and in compliance with local, national and international laws, noting any permits that were obtained (give the name of the issuing authority, the date of issue, and any identifying information).* |
| Disturbance | *Describe any disturbance caused by the study and how it was minimized.* |

# Reporting for specific materials, systems and methods

We require information from authors about some types of materials, experimental systems and methods used in many studies. Here, indicate whether each material, system or method listed is relevant to your study. If you are not sure if a list item applies to your research, read the appropriate section before selecting a response.

### Materials & experimental systems

| n/a | Involved in the study | |
|---|---|---|
| ☐ | ☒ | Antibodies |
| ☐ | ☒ | Eukaryotic cell lines |
| ☒ | ☐ | Palaeontology and archaeology |
| ☒ | ☐ | Animals and other organisms |
| ☒ | ☐ | Clinical data |
| ☒ | ☐ | Dual use research of concern |
| ☒ | ☐ | Plants |

### Methods

| n/a | Involved in the study | |
|---|---|---|
| ☒ | ☐ | ChIP-seq |
| ☐ | ☒ | Flow cytometry |
| ☒ | ☐ | MRI-based neuroimaging |

## Antibodies

| | |
|---|---|
| Antibodies used | Antigen/Marker Fluorophore Supplier #Catalog<br>CD3 APC BioLegend #300412<br>PD-1 PE-Cy7 BioLegend #367414<br>CD69 PerCP-Cy5.5 BioLegend #310926<br>CD66b AF700 BioLegend #305114<br>PD-L1 BV605 BioLegend #329724<br>CD123 PE BD Biosciences #554529<br>CD64 BV421 BioLegend #305020<br>IFN-γ PE BioLegend #506507<br><br>Antibodies were used at a dilution of 1:100 for flow staining. |
| Validation | Flow cytometry antibodies were used according to the manufacturer's instructions. All antibodies were validated for flow cytometry analysis by the manufacturer, and the expression of CD123, CD64 and PD-L1 on neutrophils was confirmed using the corresponding isotype controls. |

## Eukaryotic cell lines

Policy information about cell lines and Sex and Gender in Research

| | |
|---|---|
| Cell line source(s) | Human embryonic kidney (HEK) 293FT cells were purchased from Sigma (#12022001). |
| Authentication | The HEK293FT cell line was authenticated by the manufacturer using short tandem repeat (STR) profiling. |
| Mycoplasma contamination | Cells were tested to be negative for mycoplasma contamination. |
| Commonly misidentified lines (See ICLAC register) | Not applicable |

## Palaeontology and Archaeology

| | |
|---|---|
| Specimen provenance | *Provide provenance information for specimens and describe permits that were obtained for the work (including the name of the issuing authority, the date of issue, and any identifying information). Permits should encompass collection and, where applicable, export.* |
| Specimen deposition | *Indicate where the specimens have been deposited to permit free access by other researchers.* |
| Dating methods | *If new dates are provided, describe how they were obtained (e.g. collection, storage, sample pretreatment and measurement), where they were obtained (i.e. lab name), the calibration program and the protocol for quality assurance OR state that no new dates are provided.* |

☐ Tick this box to confirm that the raw and calibrated dates are available in the paper or in Supplementary Information.

| | |
|---|---|
| Ethics oversight | *Identify the organization(s) that approved or provided guidance on the study protocol, OR state that no ethical approval or guidance was required and explain why not.* |

Note that full information on the approval of the study protocol must also be provided in the manuscript.

## Animals and other research organisms

Policy information about studies involving animals; ARRIVE guidelines recommended for reporting animal research, and Sex and Gender in Research

| | |
|---|---|
| Laboratory animals | *For laboratory animals, report species, strain and age OR state that the study did not involve laboratory animals.* |
| Wild animals | *Provide details on animals observed in or captured in the field; report species and age where possible. Describe how animals were caught and transported and what happened to captive animals after the study (if killed, explain why and describe method; if released, say where and when) OR state that the study did not involve wild animals.* |
| Reporting on sex | *Indicate if findings apply to only one sex; describe whether sex was considered in study design, methods used for assigning sex. Provide data disaggregated for sex where this information has been collected in the source data as appropriate; provide overall numbers in this Reporting Summary. Please state if this information has not been collected. Report sex-based analyses where performed, justify reasons for lack of sex-based analysis.* |
| Field-collected samples | *For laboratory work with field-collected samples, describe all relevant parameters such as housing, maintenance, temperature, photoperiod and end-of-experiment protocol OR state that the study did not involve samples collected from the field.* |
| Ethics oversight | *Identify the organization(s) that approved or provided guidance on the study protocol, OR state that no ethical approval or guidance was required and explain why not.* |

Note that full information on the approval of the study protocol must also be provided in the manuscript.

## Clinical data

Policy information about clinical studies

All manuscripts should comply with the ICMJE guidelines for publication of clinical research and a completed CONSORT checklist must be included with all submissions.

| | |
|---|---|
| Clinical trial registration | *Provide the trial registration number from ClinicalTrials.gov or an equivalent agency.* |
| Study protocol | *Note where the full trial protocol can be accessed OR if not available, explain why.* |
| Data collection | *Describe the settings and locales of data collection, noting the time periods of recruitment and data collection.* |
| Outcomes | *Describe how you pre-defined primary and secondary outcome measures and how you assessed these measures.* |

## Dual use research of concern

Policy information about dual use research of concern

### Hazards

Could the accidental, deliberate or reckless misuse of agents or technologies generated in the work, or the application of information presented in the manuscript, pose a threat to:

| No | Yes | |
|---|---|---|
| [x] | [ ] | Public health |
| [x] | [ ] | National security |
| [x] | [ ] | Crops and/or livestock |
| [x] | [ ] | Ecosystems |
| [x] | [ ] | Any other significant area |

### Experiments of concern

Does the work involve any of these experiments of concern:

| No | Yes | |
|---|---|---|
| [x] | [ ] | Demonstrate how to render a vaccine ineffective |
| [x] | [ ] | Confer resistance to therapeutically useful antibiotics or antiviral agents |
| [x] | [ ] | Enhance the virulence of a pathogen or render a nonpathogen virulent |
| [x] | [ ] | Increase transmissibility of a pathogen |
| [x] | [ ] | Alter the host range of a pathogen |
| [x] | [ ] | Enable evasion of diagnostic/detection modalities |
| [x] | [ ] | Enable the weaponization of a biological agent or toxin |
| [x] | [ ] | Any other potentially harmful combination of experiments and agents |

## Plants

Seed stocks: *Report on the source of all seed stocks or other plant material used. If applicable, state the seed stock centre and catalogue number. If plant specimens were collected from the field, describe the collection location, date and sampling procedures.*

Novel plant genotypes: *Describe the methods by which all novel plant genotypes were produced. This includes those generated by transgenic approaches, gene editing, chemical/radiation-based mutagenesis and hybridization. For transgenic lines, describe the transformation method, the number of independent lines analyzed and the generation upon which experiments were performed. For gene-edited lines, describe the editor used, the endogenous sequence targeted for editing, the targeting guide RNA sequence (if applicable) and how the editor was applied.*

Authentication: *Describe any authentication procedures for each seed stock used or novel genotype generated. Describe any experiments used to assess the effect of a mutation and, where applicable, how potential secondary effects (e.g. second site T-DNA insertions, mosiacism, off-target gene editing) were examined.*

## ChIP-seq

### Data deposition

[ ] Confirm that both raw and final processed data have been deposited in a public database such as GEO.

[ ] Confirm that you have deposited or provided access to graph files (e.g. BED files) for the called peaks.

Data access links
*May remain private before publication.*: *For "Initial submission" or "Revised version" documents, provide reviewer access links. For your "Final submission" document, provide a link to the deposited data.*

Files in database submission: *Provide a list of all files available in the database submission.*

Genome browser session
(e.g. UCSC): *Provide a link to an anonymized genome browser session for "Initial submission" and "Revised version" documents only, to enable peer review. Write "no longer applicable" for "Final submission" documents.*

### Methodology

Replicates: *Describe the experimental replicates, specifying number, type and replicate agreement.*

| | |
|---|---|
| Sequencing depth | *Describe the sequencing depth for each experiment, providing the total number of reads, uniquely mapped reads, length of reads and whether they were paired- or single-end.* |
| Antibodies | *Describe the antibodies used for the ChIP-seq experiments; as applicable, provide supplier name, catalog number, clone name, and lot number.* |
| Peak calling parameters | *Specify the command line program and parameters used for read mapping and peak calling, including the ChIP, control and index files used.* |
| Data quality | *Describe the methods used to ensure data quality in full detail, including how many peaks are at FDR 5% and above 5-fold enrichment.* |
| Software | *Describe the software used to collect and analyze the ChIP-seq data. For custom code that has been deposited into a community repository, provide accession details.* |

# Flow Cytometry

## Plots

Confirm that:

- [x] The axis labels state the marker and fluorochrome used (e.g. CD4-FITC).
- [x] The axis scales are clearly visible. Include numbers along axes only for bottom left plot of group (a 'group' is an analysis of identical markers).
- [x] All plots are contour plots with outliers or pseudocolor plots.
- [x] A numerical value for number of cells or percentage (with statistics) is provided.

## Methodology

| | |
|---|---|
| Sample preparation | Neutrophils and T cells isolated from sepsis patients and healthy donors, with or without co-culture, were stained with specific antibodies and analysed by flow cytometry as described in the Methods section. CD4+ and CD8+ T cells were separated from PBMCs by positive selection with magnetic MicroBeads (Miltenyi Biotec) following the manufacturer's instructions. Neutrophils were extracted from whole blood from sepsis patients or healthy donors using EasySep HLA Chimerism Whole Blood CD66b positive selection kit (STEMCELL) as per manufacturer's instructions. |
| Instrument | LSRFortessa X-20 (BD Biosciences) flow cytometer. |
| Software | FlowJo software (v10.10). |
| Cell population abundance | The purity of isolated cells exceeded 95% as determined by post-extraction analysis. |
| Gating strategy | Gating strategies are described in the Supplementary Information. Briefly, viable cells were identified using the LIVE/DEAD™ Fixable Green Dead Cell Stain Kit (Thermo Fisher). T cells were gated based on CD3 expression, and neutrophils were gated using the CD66b marker. |

- [x] Tick this box to confirm that a figure exemplifying the gating strategy is provided in the Supplementary Information.

# Magnetic resonance imaging

## Experimental design

| | |
|---|---|
| Design type | *Indicate task or resting state; event-related or block design.* |
| Design specifications | *Specify the number of blocks, trials or experimental units per session and/or subject, and specify the length of each trial or block (if trials are blocked) and interval between trials.* |
| Behavioral performance measures | *State number and/or type of variables recorded (e.g. correct button press, response time) and what statistics were used to establish that the subjects were performing the task as expected (e.g. mean, range, and/or standard deviation across subjects).* |

## Acquisition

| | |
|---|---|
| Imaging type(s) | *Specify: functional, structural, diffusion, perfusion.* |
| Field strength | *Specify in Tesla* |
| Sequence & imaging parameters | *Specify the pulse sequence type (gradient echo, spin echo, etc.), imaging type (EPI, spiral, etc.), field of view, matrix size, slice thickness, orientation and TE/TR/flip angle.* |
| Area of acquisition | *State whether a whole brain scan was used OR define the area of acquisition, describing how the region was determined.* |

Diffusion MRI ☐ Used ☐ Not used

## Preprocessing

| | |
|---|---|
| Preprocessing software | *Provide detail on software version and revision number and on specific parameters (model/functions, brain extraction, segmentation, smoothing kernel size, etc.).* |
| Normalization | *If data were normalized/standardized, describe the approach(es): specify linear or non-linear and define image types used for transformation OR indicate that data were not normalized and explain rationale for lack of normalization.* |
| Normalization template | *Describe the template used for normalization/transformation, specifying subject space or group standardized space (e.g. original Talairach, MNI305, ICBM152) OR indicate that the data were not normalized.* |
| Noise and artifact removal | *Describe your procedure(s) for artifact and structured noise removal, specifying motion parameters, tissue signals and physiological signals (heart rate, respiration).* |
| Volume censoring | *Define your software and/or method and criteria for volume censoring, and state the extent of such censoring.* |

## Statistical modeling & inference

| | |
|---|---|
| Model type and settings | *Specify type (mass univariate, multivariate, RSA, predictive, etc.) and describe essential details of the model at the first and second levels (e.g. fixed, random or mixed effects; drift or auto-correlation).* |
| Effect(s) tested | *Define precise effect in terms of the task or stimulus conditions instead of psychological concepts and indicate whether ANOVA or factorial designs were used.* |

Specify type of analysis: ☐ Whole brain ☐ ROI-based ☐ Both

| | |
|---|---|
| Statistic type for inference<br>(See Eklund et al. 2016) | *Specify voxel-wise or cluster-wise and report all relevant parameters for cluster-wise methods.* |
| Correction | *Describe the type of correction and how it is obtained for multiple comparisons (e.g. FWE, FDR, permutation or Monte Carlo).* |

## Models & analysis

| n/a | Involved in the study |
|---|---|
| ☐ | ☐ Functional and/or effective connectivity |
| ☐ | ☐ Graph analysis |
| ☐ | ☐ Multivariate modeling or predictive analysis |

| | |
|---|---|
| Functional and/or effective connectivity | *Report the measures of dependence used and the model details (e.g. Pearson correlation, partial correlation, mutual information).* |
| Graph analysis | *Report the dependent variable and connectivity measure, specifying weighted graph or binarized graph, subject- or group-level, and the global and/or node summaries used (e.g. clustering coefficient, efficiency, etc.).* |
| Multivariate modeling and predictive analysis | *Specify independent variables, features extraction and dimension reduction, model, training and evaluation metrics.* |
